# Defining the Spectrum, Treatment and Outcome of Patients With Genetically Confirmed Gorlin Syndrome From the HIT-MED Cohort

**DOI:** 10.3389/fonc.2021.756025

**Published:** 2021-11-23

**Authors:** Katja Kloth, Denise Obrecht, Dominik Sturm, Torsten Pietsch, Monika Warmuth-Metz, Brigitte Bison, Martin Mynarek, Stefan Rutkowski

**Affiliations:** ^1^ Department of Pediatric Hematology and Oncology, University Medical Center Hamburg-Eppendorf, Hamburg, Germany; ^2^ Hopp Children’s Cancer Center (KiTZ) Heidelberg, Heidelberg, Germany; ^3^ Division of Pediatric Glioma Research, German Cancer Research Center (DKFZ) Heidelberg, Heidelberg, Germany; ^4^ Department of Pediatric Oncology, Hematology, and Immunology, Heidelberg University Hospital, Heidelberg, Germany; ^5^ Department of Neuropathology, Deutsche Gesellschaft für Neuropathologie und Neuroanatomie (DGNN) Brain Tumor Reference Center, Bonn, Germany; ^6^ Institute of Diagnostic and Interventional Neuroradiology, University Hospital Wuerzburg, Wuerzburg, Germany; ^7^ Department of Diagnostic and Interventional Neuroradiology, University Hospital Augsburg, Augsburg, Germany

**Keywords:** Gorlin, *PTCH1*, *SUFU*, medulloblastoma, childhood cancer predisposition syndrome

## Abstract

Gorlin syndrome is a genetic condition associated with the occurrence of SHH activated medulloblastoma, basal cell carcinoma, macrocephaly and other congenital anomalies. It is caused by heterozygous pathogenic variants in *PTCH1* or *SUFU*. In this study we included 16 patients from the HIT2000, HIT2000interim, I-HIT-MED, observation registry and older registries such as HIT-SKK87, HIT-SKK92 (1987 – 2020) with genetically confirmed Gorlin syndrome, harboring 10 *PTCH1* and 6 *SUFU* mutations. Nine patients presented with desmoplastic medulloblastomas (DMB), 6 with medulloblastomas with extensive nodularity (MBEN) and one patient with classic medulloblastoma (CMB); all tumors affected the cerebellum, vermis or the fourth ventricle. SHH activation was present in all investigated tumors (14/16); DNA methylation analysis (when available) classified 3 tumors as iSHH-I and 4 tumors as iSHH-II. Age at diagnosis ranged from 0.65 to 3.41 years. All but one patient received chemotherapy according to the HIT-SKK protocol. Ten patients were in complete remission after completion of primary therapy; four subsequently presented with PD. No patient received radiotherapy during initial treatment. Five patients acquired additional neoplasms, namely basal cell carcinomas, odontogenic tumors, ovarian fibromas and meningioma. Developmental delay was documented in 5/16 patients. Overall survival (OS) and progression-free survival (PFS) between patients with *PTCH1* or *SUFU* mutations did not differ statistically (10y-OS 90% *vs*. 100%, p=0.414; 5y-PFS 88.9% ± 10.5% *vs*. 41.7% ± 22.2%, p=0.139). Comparing the Gorlin patients to all young, SHH activated MBs in the registries (10y-OS 93.3% ± 6.4% *vs*. 92.5% ± 3.3%, p=0.738; 10y-PFS 64.9%+-16.7% *vs*. 83.8%+-4.5%, p=0.228) as well as comparing Gorlin M0 SKK-treated patients to all young, SHH activated, M0, SKK-treated MBs in the HIT-MED database did not reveal significantly different clinical outcomes (10y-OS 88.9% ± 10.5% *vs*. 88% ± 4%, p=0.812; 5y-PFS 87.5% ± 11.7% *vs*. 77.7% ± 5.1%, p=0.746). Gorlin syndrome should be considered in young children with SHH activated medulloblastoma, especially DMB and MBEN but cannot be ruled out for CMB. Survival did not differ to patients with SHH-activated medulloblastoma with unknown germline status or between *PTCH1* and *SUFU* mutated patients. Additional neoplasms, especially basal cell carcinomas, need to be expected and screened for. Genetic counselling should be provided for families with young medulloblastoma patients with SHH activation.

## Introduction

Tumor predisposition syndromes are hereditary diseases causing a higher risk to develop certain benign or malignant neoplasms in adults and children (1, 2). In adults, the percentage of malignancies attributed to causative genetic alterations is said to be 5-10% (1, 3). In childhood, there is an overlapping but different spectrum of syndromes associated with tumor predisposition, like Li-Fraumeni syndrome, Gorlin syndrome, Fanconi anemia, tuberous sclerosis, neurofibromatosis, Cowden syndrome, APC related adenomatous polyposis, Beckwith-Wiedemann syndrome, etc (1, 4, 5).

Genetic cancer predisposition rates for children have lately been reanalyzed after the discovery of now more than 100 associated genes, and causative germline variants were identified in up to 8.5% of affected individuals (6–8), most of them presenting with an unremarkable family history (8). However, certain entities are more frequently assessed and directed towards genetic testing, making the diagnosis of a hereditary tumor syndrome more likely, e.g. in younger women diagnosed with breast or ovarian cancer or very young children with Wilms or adrenal cortical tumors in (1, 3).

Gorlin, Li-Fraumeni and rhabdoid tumor predisposition syndrome (RTPS) as well as neurofibromatosis type 1 and 2, von Hippel-Lindau syndrome and tuberous sclerosis complex (TSC) are some of the syndromes associated with an increased risk for childhood-onset brain tumors (9, 10). Brain tumors are the most common solid malignancies in childhood with medulloblastomas being the second most frequent entity constituting nearly 20% of all pediatric brain tumors (11–13). Medulloblastomas arise from the cerebellum, vermis or fourth ventricle/posterior fossa and split up in 4 different molecular subgroups: wingless (WNT)-activated (TP53wt), wingless (WNT)-activated (TP53mut), sonic hedgehog (SHH)-activated (TP53wt), MB without WNT/SHH activation (Group 3 or Group 4 (G3/4)) as defined in the 2021 WHO classification (14, 15). They are further characterized by their varying origins, molecular drivers, demographics and clinical outcomes (16, 17).

SHH-activated medulloblastomas (SHH-MB) account for approximately 25% of all medulloblastomas. Almost all SHH-MB contain at least one driver event, most frequently affecting *PTCH1, SUFU, TP53* or *SMO, KMT2D/2C*, *HAT, GPR161* and *ELP1;* with germline mutations in *TP53* and *ELP1* mostly identified in older pediatric medulloblastoma patients (16, 18–20). The number of damaging germline mutations identified is highest in this subgroup; *MYC* or *MYCN* genes are also regularly amplified (16, 21).

SHH-MB occur at two age peaks, in infancy/young childhood and adulthood - with 50% of the affected children being diagnosed before the age of 5 years. At the time of diagnosis 30-40% of patients present with metastatic disease; the 5 year overall survival for this group is only 66% with many long term survivors facing treatment-related neurological adverse effects (22, 23). Median survival time for relapsed disease is still less than 1 year (24).

Patients under the age of 3 will preferably be treated by systemic interval chemotherapy after gross resection of the tumor. Depending on the risk stratification, irradiation can be omitted during initial therapy or delayed until the child is older (or a relapse occurs) (25). Delaying or omitting craniospinal radiotherapy is especially successful in children with non-metastatic disease presenting with desmoplastic or extensive nodular histology which is a strong independent favorable prognostic factor compared to classical MB (26–29). A chemotherapy-only approach (e.g. HIT-SKK regime) is especially favorable in young Gorlin patients where a radiotherapy-sparing treatment option is important to prevent the occurrence of secondary neoplasms like basal cell carcinomas (BCC) (26, 27, 30–34). In line with this, the choice of the primary treatment with the highest possible chance to avoid relapse and consecutively radiotherapy is key (26, 27, 35, 36).

Tumor predisposition syndromes - like *PTCH1* or *SUFU* associated Gorlin and *TP53* associated Li-Fraumeni syndrome - affect approximately 7-8% of children with childhood/adolescent cancers and 5-6% of medulloblastoma patients with the highest prevalence of 14-20% for germline mutations in the SHH-MB subgroup (16, 19, 30, 37). Gorlin syndrome is diagnosed at a prevalence of approximately 1:30.000 - 60.000 (38, 39).

The risk for developing a medulloblastoma in Gorlin patients is estimated at 2-5% with a male predominance of approximately 3:1, usually occurring in the first 3 years of life (16, 30). The syndrome was first described in 1960 by Gorlin and Goltz. They initially described a subgroup of patients with basal cell carcinomas, jaw cysts and congenital rib anomalies (40). Subsequently, causative mutations in the genes *PTCH1* and *SUFU* were identified (9, 41). Recently, potentially disease causing heterozygous *PTCH2* variants have been identified in patients with a milder Gorlin associated phenotype and controversially discussed but this gene has not yet been included in routine testing for Gorlin syndrome (42, 43). Smoothened (*SMO*) is another candidate gene frequently harboring somatic mutations in medulloblastoma tumors but also affecting the germline in adult medulloblastoma patients, potentially opening the door for target therapies such as SMO inhibitors (31, 44).

The *PTCH1* or *SUFU* associated Gorlin syndrome follows an autosomal dominant inheritance pattern; up to 80% of the mutations seem to be familial with a sporadic *de novo* event occurring in 20-30% (38, 39). Offspring of an affected individual will inherit the pathogenic variant in 50%. Penetrance is described to be almost 100% with a highly variable expression (32, 39).

Approximately 60% of Gorlin patients present with typical phenotypic features such as macrocephaly, frontal bossing, coarse facial features, palmar/plantar pits and/or skeletal abnormalities, e.g. of the ribs and vertebrae. Some degree of motor delay is often described, although this is almost always temporary. Global developmental delay is not routinely associated with Gorlin syndrome (33).

Neoplasms in patients with Gorlin syndrome include the typical basal cell nevi/carcinomas (90-100%), jaw keratocysts (90%), cardiac and/or ovarian fibromas (2-20%) and medulloblastomas (5%); most commonly the desmoplastic subtype.

Medulloblastomas occur significantly more often in patients with pathogenic *SUFU* variants (33%) than in those harboring *PTCH1* variants (<2%) (45, 46). Additionally, the risk for radiation-induced meningioma is significantly higher in *SUFU* mutated patients (47). General life expectancy is not reduced in Gorlin patients (33).

## Materials and Methods

The international HIT-SKK87, HIT-SKK92, HIT2000interim, HIT2000, I-HIT-MED and observation registries were retrospectively screened for patients with suspected or genetically confirmed Gorlin syndrome. A prospective screening for Gorlin syndrome was not part of this study. 2232 patients (0 – 18y) with medulloblastomas were diagnosed between 1987and 2020 and included in one of the registries mentioned above. Out of those 2232 patients, 323 were histologically classified as DMB (0.2y – 17.8y) or MBEN (0.2 – 4.1y) and 1779 as CMB (0.0 – 17.9y) by local pathologists and/or central neuropathological review since 1994 at the Brain Tumor Reference Center of the DGNN at the Institute of Neuropathology, University of Bonn Medical Center, Germany (n=1475). Patients were included in this study if the diagnosis of Gorlin syndrome was genetically confirmed by germline genetic testing and defined as the Gorlin Cohort.

SHH activation was tested by immunohistochemistry or DNA methylation analysis as described previously (48–50).

To form the Comparative Cohort all patients from the existing registries (see above) were screened for age < 3.5 years at the time of diagnosis and SHH activation. To form the M0 Comparative Cohort the patients were screened for M0 status at the time of diagnosis. Gorlin syndrome was not ruled out systematically by genetic testing in all of these patients.

Progression-free survival (PFS) was defined as the time from surgery to first progression (progression or relapse) or date of last follow-up. Overall survival (OS) was defined as the time from surgery to reported date of death or until a certain point in time within the follow-up for a specific patient, e.g. 3 year-OS or 5 year-OS. Survival of patient groups was compared by log-rank test and Kaplan-Meier curves were constructed.

All examinations were carried out on the basis and according to the legal requirements of the revised Declaration of Helsinki of the World Medical Association in 1983. Informed consent was given at study inclusion by the parents or adolescent patients themselves. Corresponding demographic and clinical data were extracted from the existing registry database (see above).

## Results

### Patients’ Characteristics

**Figure 1:**
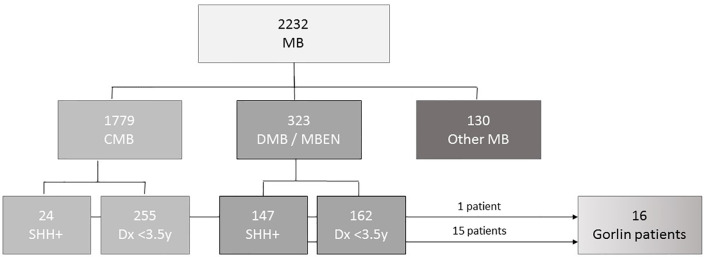
CONSORT diagram of the cohorts’ patient characterics.

As of November 2020 there were 2232 patients <18y with medulloblastoma registered in the current and former HIT registries, namely I-HIT-MED (NCT02417324), HIT2000 interim registry (NCT02238899), HIT2000 (NCT00303810), HIT-SKK87 and HIT-SKK92 (26, 27). 323 of these patients were diagnosed with desmoplastic medulloblastoma (DMB) or medulloblastomas with extensive nodularity (MBEN). For 147 patients SHH activation was observed by molecular neuropathological assessment. 162 patients in this cohort were diagnosed when 3.5 years old or younger. 94 of those presented with SHH activation; SHH activation was not assessed in the remaining patients. A total of 1779 patients presented with CMB. Out of the 640 that underwent DNA methylation analysis, 24 patients presented with SHH activation. 8 of these were diagnosed aged 3.5 years or younger (see **Figure 1**).

### Gorlin Cohort

From our above mentioned registries, we obtained genetic confirmation of Gorlin syndrome for 16 patients. There were 8 affected females and males, respectively. In 10 patients *PTCH1* mutations were identified, while 6 patients presented with pathogenic or likely pathogenic *SUFU* variants. *PTCH1* mutations were detected in 4 females and 6 males each, *SUFU* mutations were identified in 4 females and 2 males.

Family history was positive for Gorlin syndrome in 5/16 of patients (31.3%): In one family identical twins were affected, in one family the mother and one sister were affected, in one family two cousins were affected (the parents of the patients or their siblings were not mentioned) and in another family the father was also affected. All patients with a positive family history carried *PTCH1* variants, except for the two affected cousins harboring *SUFU* mutations.

Histologically, 9 patients presented with desmoplastic medulloblastoma (DMB), 6 patients with medulloblastomas with extensive nodularity (MBEN) and 1 patient with a classic medulloblastoma (CMB). 12 were localized in the cerebellum, vermis and/or the 4^th^ ventricle while 4 were located in the cerebellar hemispheres (see **Table 1**).

**Table 1:** Patient characteristics for the Gorlin cohort (all genetically diagnosed *PTCH1* and *SUFU* associated Gorlin syndrome patients) *vs.* the Comparative cohort (all DMB/MBEN, SHH+, <3.5y).

	Gorlin cohort (all genetically confirmed Gorlin patients) n = 16	Comparative cohort (all DMB/MBEN, SHH+, <3.5y) n = 92
**Age [years]**	0.65 – 3.41	0.2 – 3.5
**Sex**		
Male	8	87
Female	8	63
**Histology**		
DMB/MBEN	9	60
MBEN	6	32
CMB	1	n/a
**Staging**		
M0	13	63
M+	3	23
n/a	0	5
R0 (no rest, <1.5cm^2^)	11	64
R+ (>1.5cm^2^)	5	24
n/a	0	4
**Initial Treatment**		
SKK therapy	15	54
Intensified Induction	0	14
Other	1	23
**Trial**		
HIT-SKK’92	1	3
HIT2000 Interim Registry	2	9
HIT2000	3	42
I-HIT-MED	8	36
Observation registry	2	2

Age at diagnosis ranged from 0.65 to 3.41 years with a median age of 0.97 years. Median age at diagnosis in the *PTCH1* mutated group was 0.9 years *vs*. 2.12 years in the *SUFU* mutated group.

Biological workup was completed in 10 patients who showed no amplification of *MYC* or *MYCN*; SHH activation was shown in 14/16 patients (8 DMBs, 5 MBENs, 1 CMB). The remainder of the patients was not screened for *MYC/MYCN* amplification or SHH activation. 4/16 patients presented with M+ status at initial diagnosis (see **Table 1**).

DNA methylation profiles were available for 8 of these patients and were further subclassified by *t*-distributed stochastic neighbor embedding (*t*-SNE) to differentiate between iSHH-I and iSHH-II (28, 35, 51, 52). It revealed SHH-I in 3 patients, SHH-II in 4 patients and 1 unclear result. Copy-number variation (CNV) analysis revealed varying chromosomal anomalies. Findings in the *PTCH1* group included loss of chromosome 9 in 2 patients and loss of 2qtel and a small deletion in 8q. Results in the *SUFU* group showed loss of chromosome 10 or 10q in 2 patients, loss of 16q, gain of chromosome 3, 4, 9, 13 and 15, gain of 3q, gain of 19q and a flat genome in 3 patients. Due to the small number of analyses, no statistically relevant differences could be detected between *PTCH1* and *SUFU* mutated patients.

4 patients were included in the MNP2.0 study which included screening for somatic and/or germline variants *via* next generation sequencing (NGS) (36): 1 patient`s somatic workup was unremarkable, 1 patient presented with a somatic *SUFU* and *KMT2D* variant, 1 patient presented with 2 somatic *SUFU* variants, 1 *PIK3CA* and 1 *GSE1* variant and in another patient a somatic *PTEN* variant was identified. 3 of these patients showed remarkable findings in the germline in the study and subsequently underwent routine germline genetic testing after genetic counseling. The patient whose somatic workup was unremarkable subsequently underwent NGS germline testing which revealed a pathogenic *SUFU* variant. The patient with the somatic *PTEN* variant subsequently underwent targeted germline testing which revealed a pathogenic *SUFU* variant.

### Dysmorphism/Accompanying Clinical Features

11 patients presented with dysmorphic or congenital anomalies at the age of diagnosis of medulloblastoma: 8 patients presented with macrocephaly; in all 11 patients other abnormalities like thoracic, vertebral or rib malformations, hypertelorism, hydrocephalus, turricephalus, craniosynostosis, strabismus, frontal bossing, hemangioma, palmoplantar pits/dents, scapula alata, pectus carinatum, optic atrophy, short stature and/or scoliosis were documented. 7 out of 10 *PTCH1* (70%) mutated patients showed phenotypic anomalies, while 4/6 patients (66.7%) with *SUFU* mutations presented with signs of dysmorphism. 2 dysmorphic patients had been diagnosed with developmental delay prior to their diagnosis of MB; 1 of them carried a *PTCH1* and the other a *SUFU* mutation. A detailed neuropsychological evaluation was not available for these patients. 5 patients showed no dysmorphic features or anomalies at the time of diagnosis of the MB, making the medulloblastoma the first symptom of the syndrome.

### Therapy

**Figure 2:**
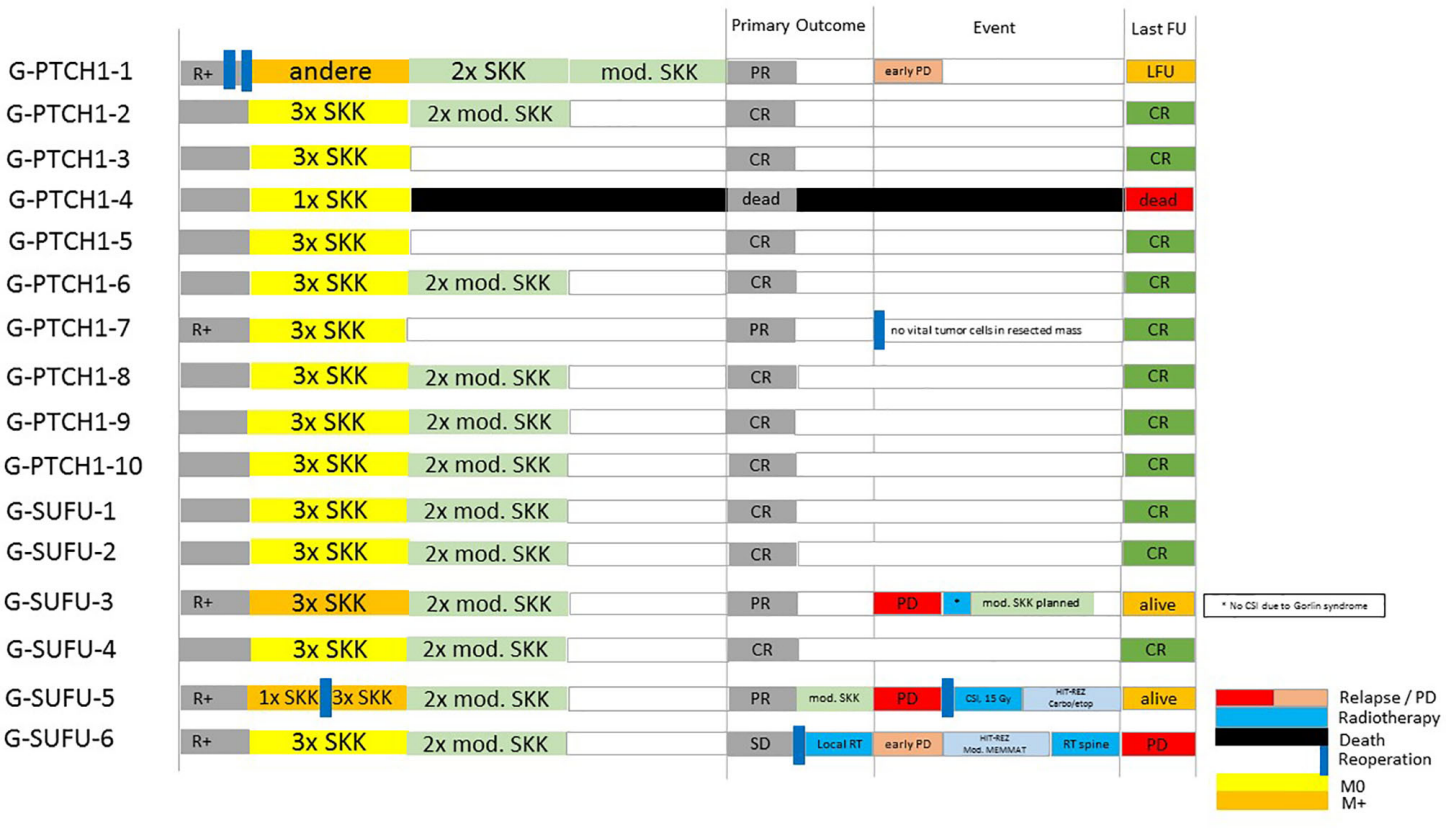
Clinical courses of patients from the Gorlin cohort.

All 16 patients received adjuvant chemotherapy following resection. 15/16 patients received the standardized HIT-SKK regime depending on the currently applicable study protocol; e.g. 3 cycles of SKK or 3 SKK cycles followed by 2 modified SKK cycles (26, 28, 53). One patient received a modified chemotherapy regime in his home country (TOT1 (CPM/VCR; CPM/VCR; CDDP/Eto), followed by 1x CPM/VCR without MTX). 13/15 patients received 3 SKK cycles, 1 patient received 4 cycles and 1 patient discontinued treatment after a resuscitation under chemotherapy resulting in hypoxic brain damage. Outcome after completion of initial treatment was CR in 10 patients, PR in 4 patients and SD in one patient (see **Figure 2**). Outcome at the last follow up (FU) was CR in 11 patients, PD in one patient and PR or SD in 2 patients. One patient had died, and one patient was lost to follow-up (LFU).

One patient with PD underwent another round of modified SKK (see **Table 1** and **Figure 2**).

Intraventricular methotrexate (26, 28) was administered in at least 12/16 patients (75%). 9 patients received the full or at least 75% of the target dosage. 3 patients received a significantly reduced amount.

0/16 patients received radiotherapy during initial treatment. 2 patients received radiotherapy because of PD or relapse: 1 patient received radiotherapy following a relapse under salvage chemotherapy after initially declining CSI. CSI was administered following resection of the recurrence and stem cell transplantation but was discontinued after 15 Gy due to the diagnosis of the pathogenic *SUFU* mutation (see **Figure 2**, patient G-SUFU-5). The second patient received local radiotherapy with 54 Gy because of PD of his residual tumor mass (initially R+) upon completing the 2^nd^ cycle of modified SKK. He progressed to M2/M3 under local radiotherapy and was started on a modified MEMMAT protocol (54). Since he showed no response, this was terminated and he is scheduled for palliative, salvage spinal radiation of the largest metastases (see **Figure 2**, patient G-SUFU-6).

Radiotherapy as a second line treatment was considered for at least one other patient due to persistence of residual tumor and metastases after the 1^st^ cycle of adjuvant SKK chemotherapy but was not administered because of the diagnosis of Gorlin syndrome.

### Relapse/Recurrence

Recurrence of the disease occurred in 4 out of 16 patients with Gorlin syndrome: 1 presented with local PD early after completion of chemotherapy (see **Figure 2**, patient G-PTCH1-1). 1 patient developed a local relapse/PD after initially presenting with PR following initial therapy (see **Figure 2**, patient G-SUFU-3). 1 patient presented with spinal metastases after initially presenting with PR following initial therapy (see **Figure 2**, patient G-SUFU-5). 1 patient presented with local PD under local radiotherapy after initially presenting with SD upon completion of primary therapy. This patient later additionally developed spinal metastases (see **Figure 2**, patient G-SUFU-6). All patients who relapsed presented with residual tumor after their initial surgery. 3 out of the 4 patients presented with metastatic disease at initial diagnosis. 3/4 of the relapsed Gorlin patients harbored *SUFU* mutations.

### Follow Up

Severe global developmental delay or developmental delay/cognitive deficits were documented in 3 patients; 2 of them carried *PTCH1* mutations, 1 patient carried a *SUFU* variant. In 3 patients motor development delay/motor deficits were documented. 2 additional patients had been diagnosed with developmental delay prior to their diagnosis of MB. In 2 patients leukencephalopathy grade I (LEP I) and neurotoxicity grade II were reported following treatment; detailed information on consecutive deficits was not available.

At the last follow up, 15 patients were recorded as active and alive. 1 patient with a *PTCH1* mutation had died of complications of hypoxic brain damage resulting from a resuscitation under chemotherapy.

### Additional Neoplasms

**Figure 3:**
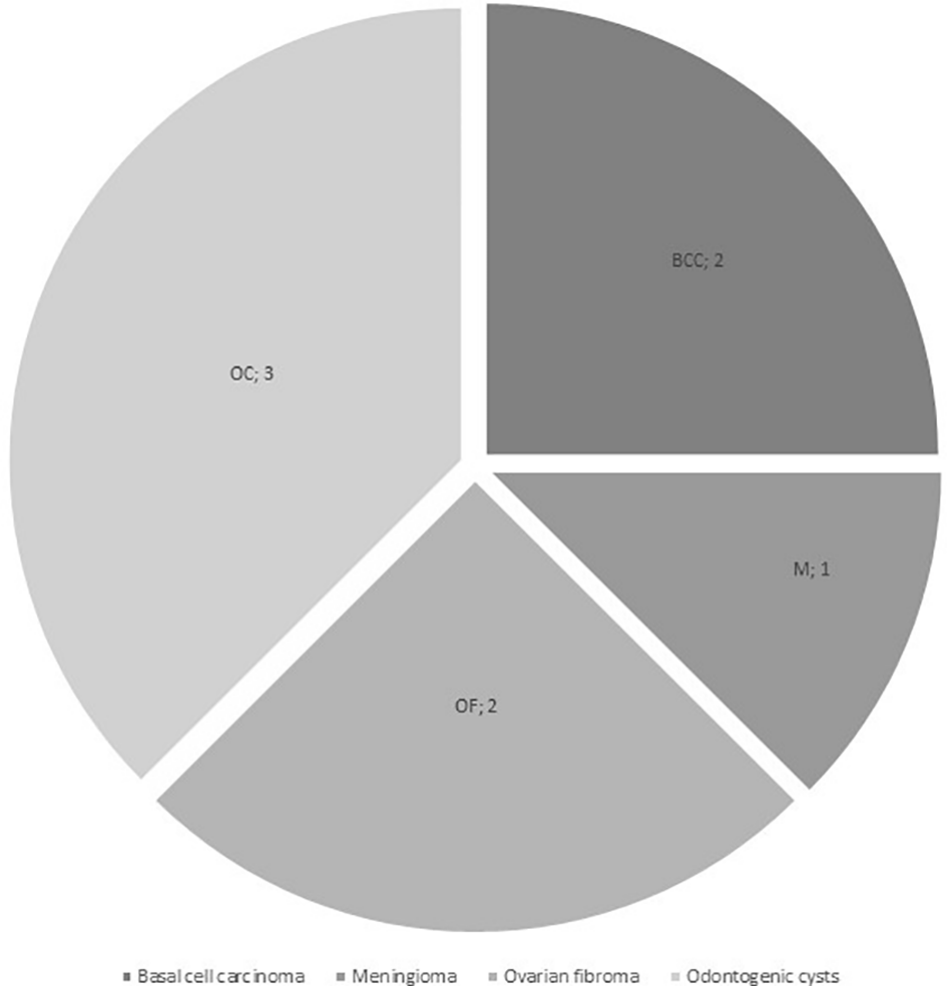
Additional neoplasms in the Gorlin Cohort (BCC, basal cell carcinoma; M, meningioma; OC, odontogenic cysts / tumors; OF, ovarian fibroma).

Additional neoplasms were reported in 5/16 patients (31.3%) from the Gorlin Cohort: one had a meningioma (M) 13 years and multiple basal cell carcinomas on head and sternum 15 years after diagnosis, one presented with multiple basal cell carcinomas of the face 9 years after treatment, odontogenic cysts at the age of 10 years and multiple ovarian fibromas (OF) on both sides at the age of 16 years, one patient had a ovarian fibroma that was operated on 11 years after initial diagnosis of the medulloblastoma and two other patients developed multiple odontogenic cysts/tumors (OC) (see **Figure 3**). All 5 patients with these additional tumors harbored *PTCH1* mutations. In comparison, all medulloblastoma patients from our current and former registries combined present with a cumulative incidence for additional/secondary neoplasms of 5.05%.

### Survival

**Figure 4.1:**
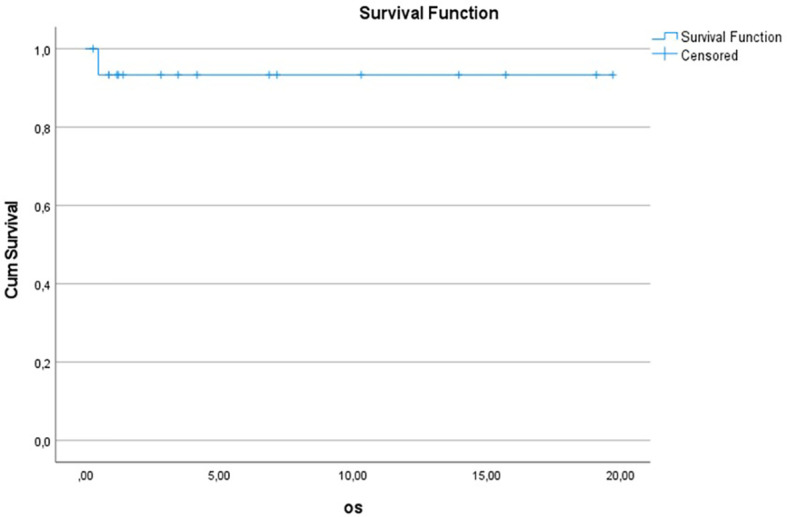
Kaplan-Meier plot of OS for all patients with Gorlin syndrome (n=16).

**Figure 4.2:**
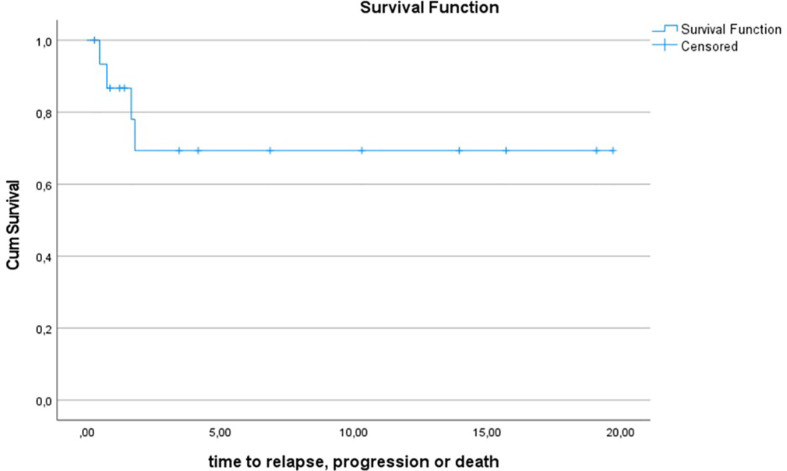
Kaplan-Meier plot PFS for all patients with Gorlin syndrome (n=16).

10y-OS for the entire Gorlin cohort was 93.3% ± 6.4%. 1/16 patients died during the follow up period (6.2%) (see **Figure 4.1**). 10y-PFS was 69.3% ± 13% (see **Figure 4.2**).

**Figure 5:**
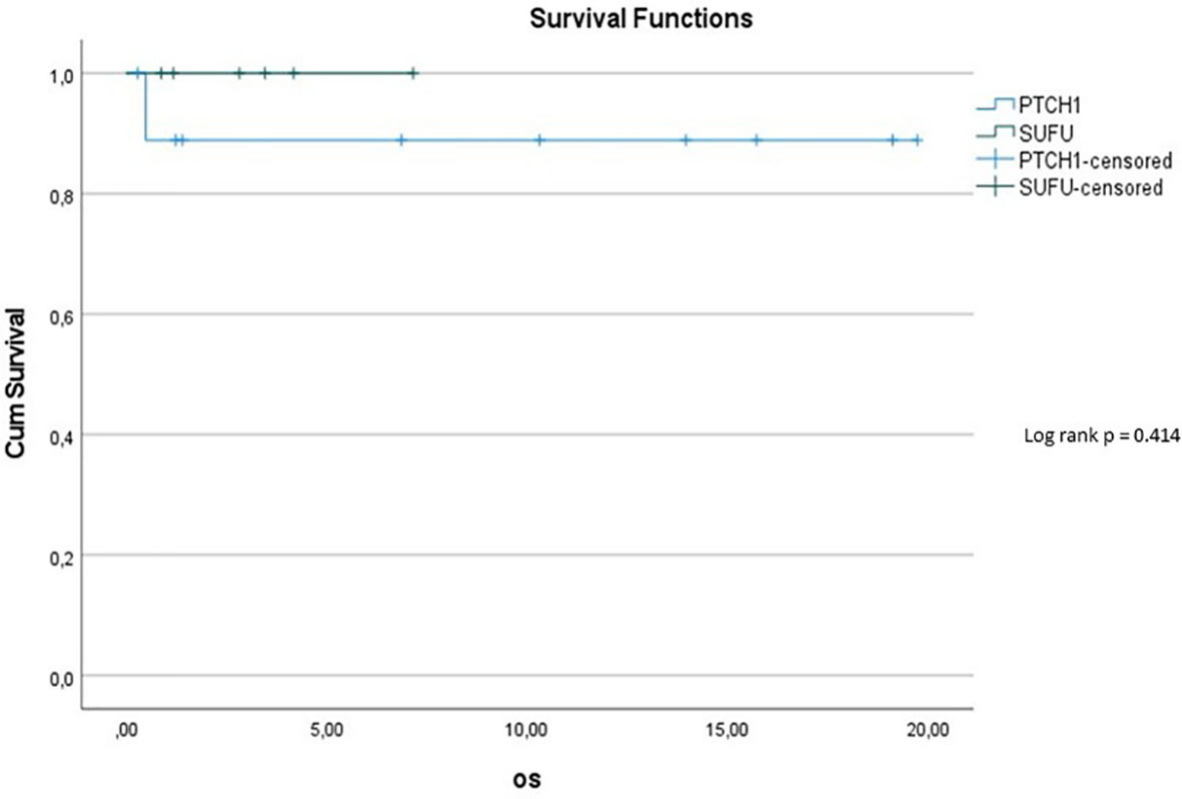
Kaplan-Meier plot for OS for patients depending on mutational status (blue = *PTCH1* mutated patients; green = *SUFU* mutated patients).

10y-OS in the *PTCH1* cohort was 90%, whereas it was 100% in the *SUFU* mutated cohort (p=0.414) (see **Figure 5**). However, the subset of patients with *PTCH1* mutations showed a median follow up of 12.1 years, while the median follow up in patients with *SUFU* mutations was 2.58 years.

**Figure 6:**
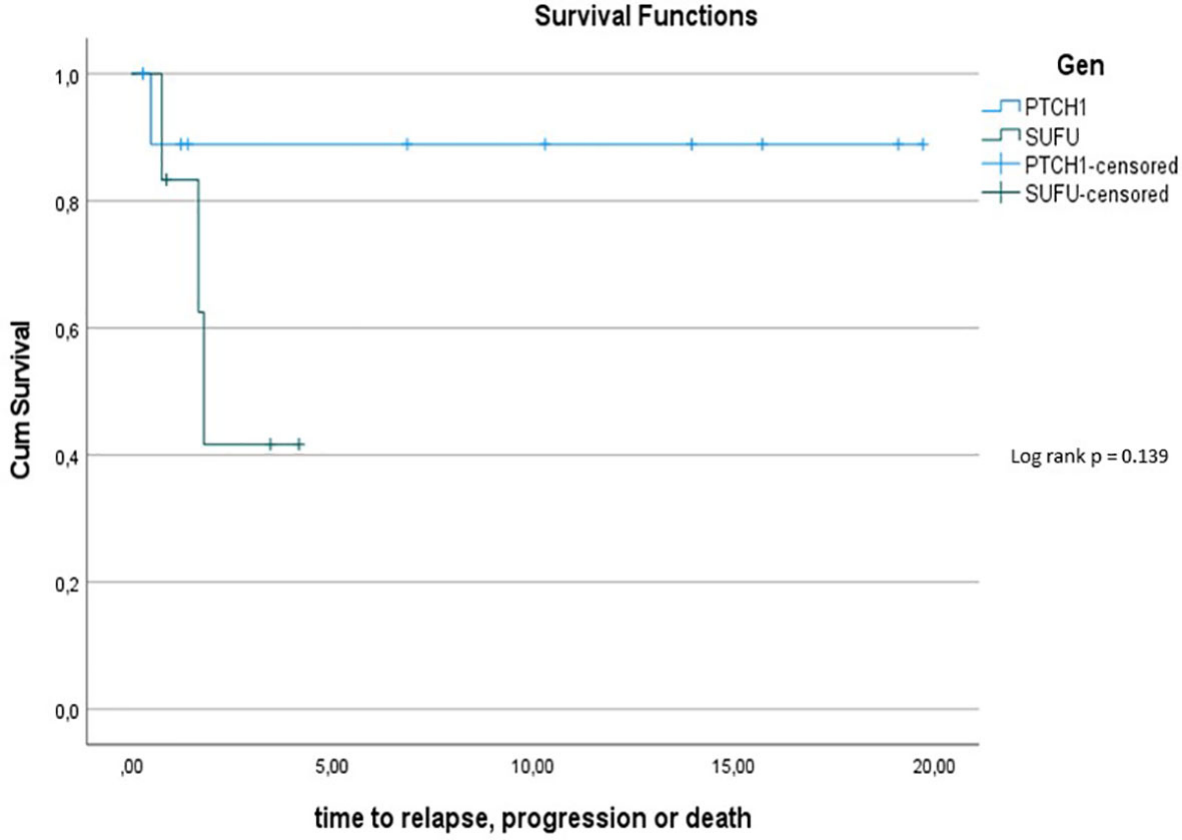
Kaplan-Meier plot for PFs for all patients depending on mutational status (blue = *PTCH1* mutated patients; green = *SUFU* mutated patients).

The 5y-PFS in the *PTCH1* mutated cohort was 88.9% ± 10.5%; the 5y-PFS in the *SUFU* mutated cohort was 41.7% ± 22.2% (p=0.139) (see **Figure 6**).

**Figure 7.1:**
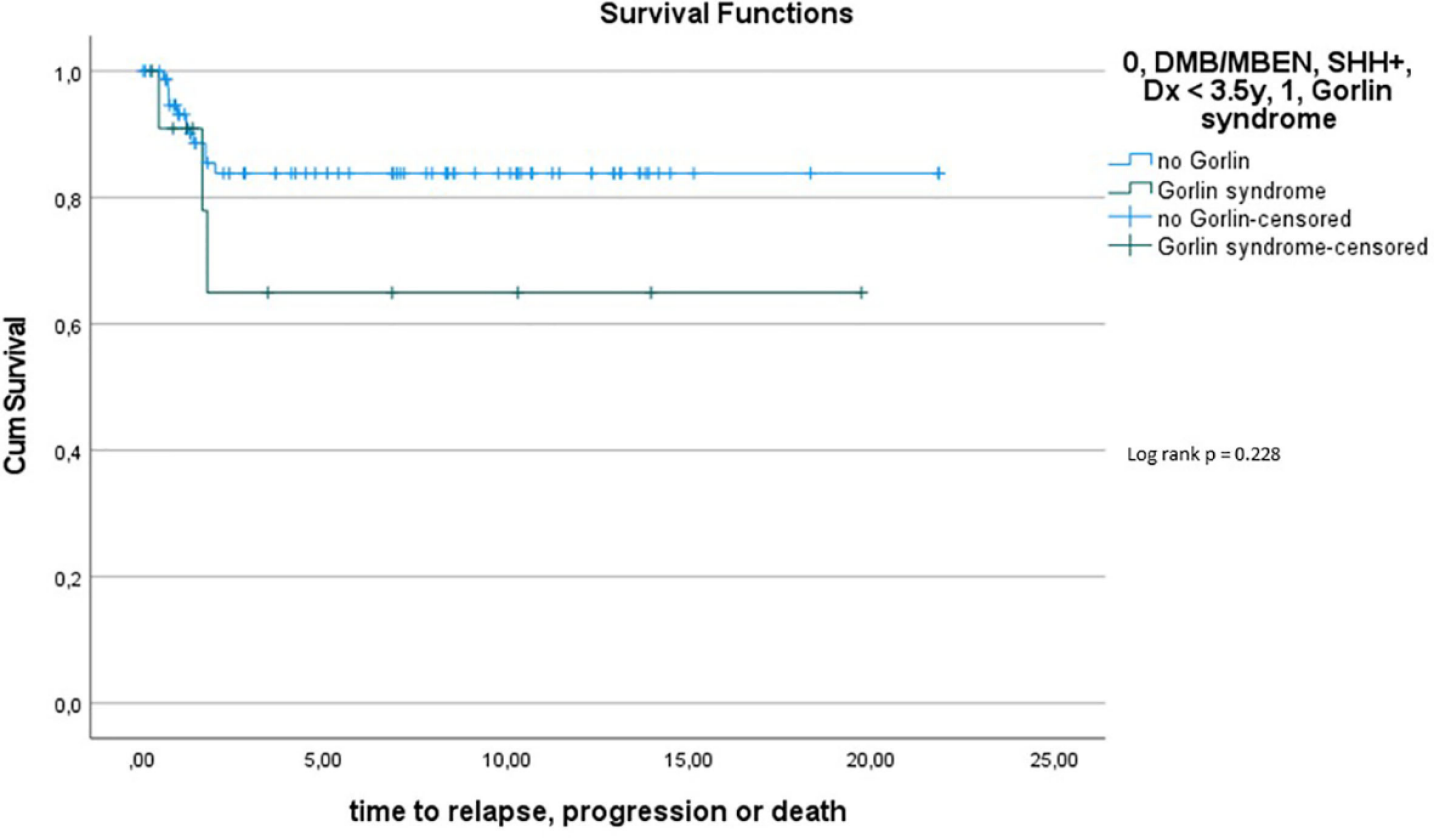
OS for all SHH +, MBEN/DMB, <3.5 *vs*. all Gorlin patients.

**Figure 7.2:**
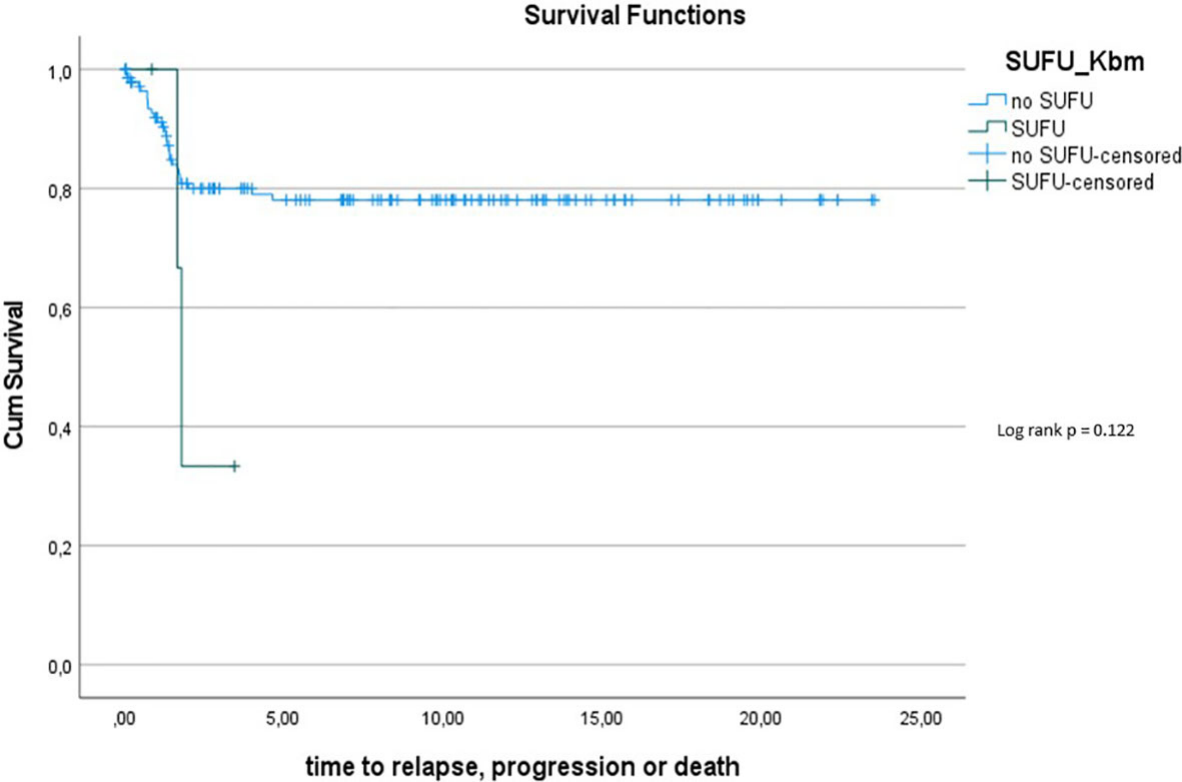
PFS for all SHH +, MBEN/DMB, <3.5 *vs*. all Gorlin patients.

**Figure 8:**
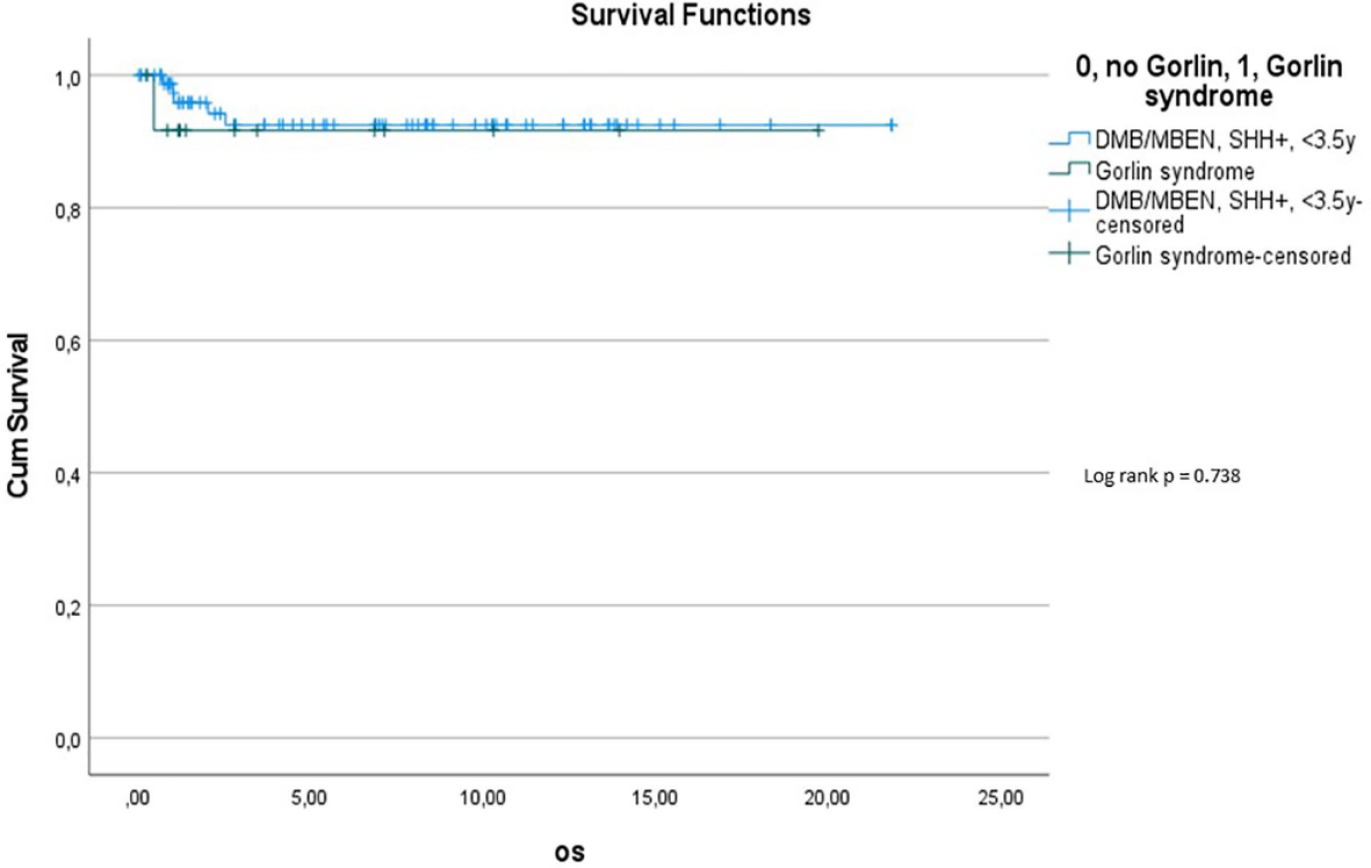
PFS for all SHH+, MBEN/DMB <3.5 *vs. SUFU* mutated Gorlin patients.

**Figure 9:**
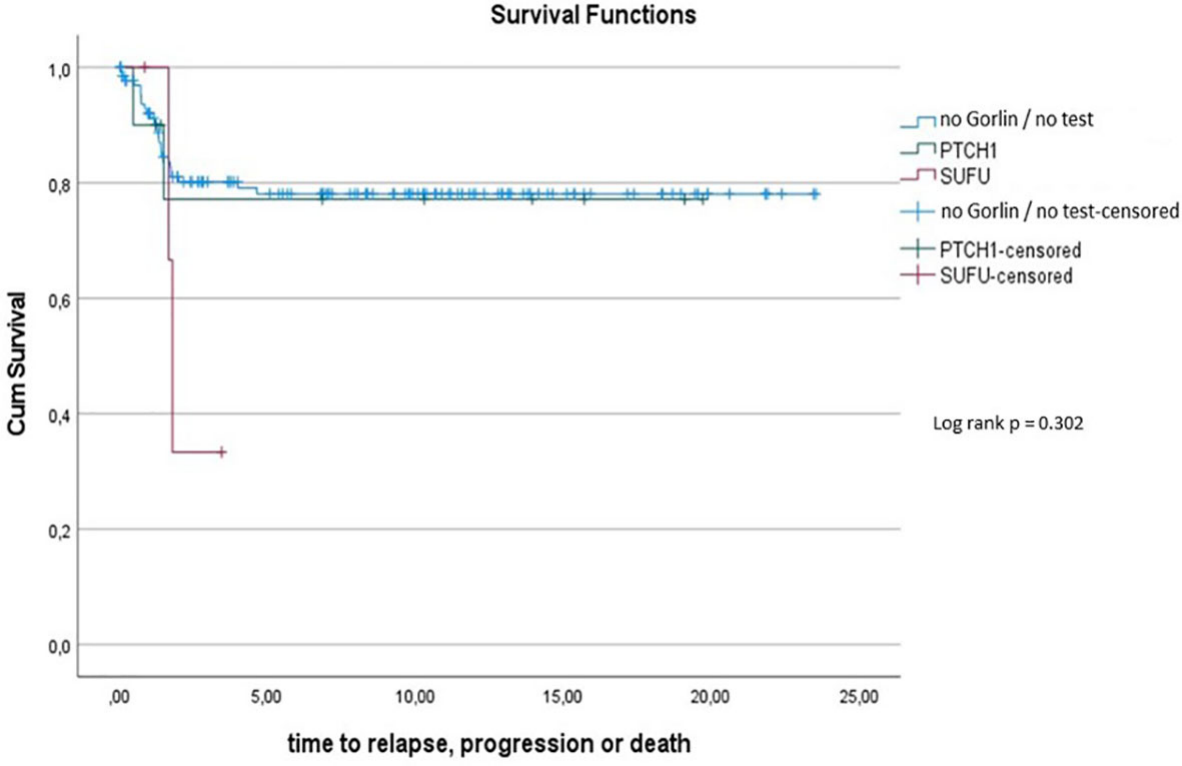
PFS for all SHH+, MBEN/DMB <3.5 *vs. PTCH1 vs. SUFU*.

Comparing survival of the Gorlin patients with the Comparative Cohort consisting of all patients from the registries with MBEN or DMB, diagnosed at 3.5y or younger, presenting with SHH activation, there was no significant difference in terms of OS (p=0.738) or PFS (p=0.228): 10y-OS in the Gorlin Cohort was 93.3% ± 6.4% *vs*. 92.5% ± 3.3% in the Comparative Cohort (p=0.738) (see **Figure 7.1**). 10y-PFS in the Gorlin Cohort was 64.9%+-16.7% *vs*. 83.8%+-4.5% in the Comparative Cohort (p=0.228) (see **Figures 7.2**, **8**, **9**).

**Figure 10:**
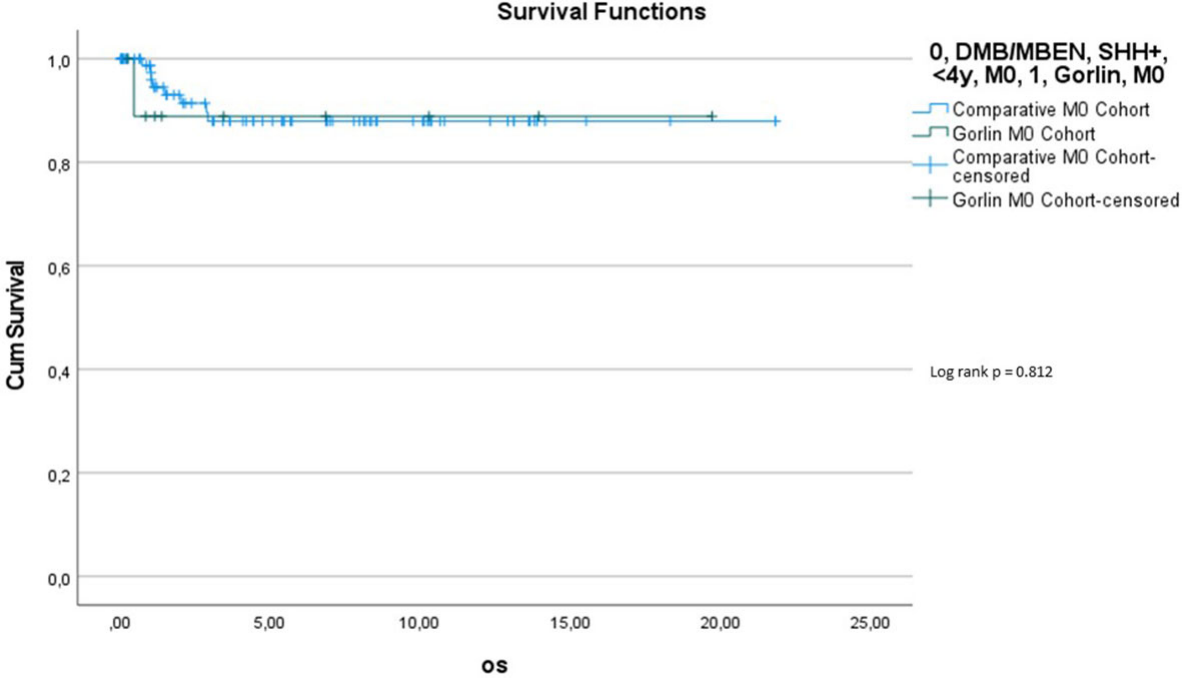
Comparison of OS (Gorlin M0 cohort *vs*. Comparative M0 Cohort).

Comparing survival of the genetically diagnosed Gorlin patients with M0 status (n=13) to the previously published SHH-activated, M0 infant medulloblastomas (n=51) (compare to (28)) (see **Table 2**), there was no significant difference in terms of OS (p=0.812): Median follow up time in the Comparative M0 cohort was 7.04 years. 10y-OS in the Gorlin M0 Cohort was 88.9% ± 10.5% *vs*. 88% ± 4% for the Comparative M0 Cohort (p=0.812) (see **Figure 10**).

**Table 2:** Patient characteristics for the Gorlin M0 Cohort (all genetically diagnosed *PTCH1* and *SUFU* associated Gorlin syndrome patients) *vs*. the Comparative M0 Cohort (all medulloblastomas (MB), SHH+, M0, <4y).

	Gorlin M0 cohort (all genetically confirmed Gorlin patients) n = 13 [this study]	Comparative M0 cohort (all MB, SHH+, M0, <4y) n = 51 [compare to Mynarek et al., J Clin Oncol., 2020]
**Age [years]**	0.65 – 3.41	0.29 – 3.79
**Sex**		
Male	7	28
Female	6	23
**Histology**		
DMB/MBEN	9	31
MBEN	3	18
CMB	1	2
**Staging**		
R0 (no rest, <1.5cm^2^)	11	43
R+ (>1.5cm^2^)	2	8
**Initial Treatment**		
SKK therapy	13	49
SKK + local RT	0	2
**Trial**		
HIT2000	4	27
Other	9	24

**Figure 11:**
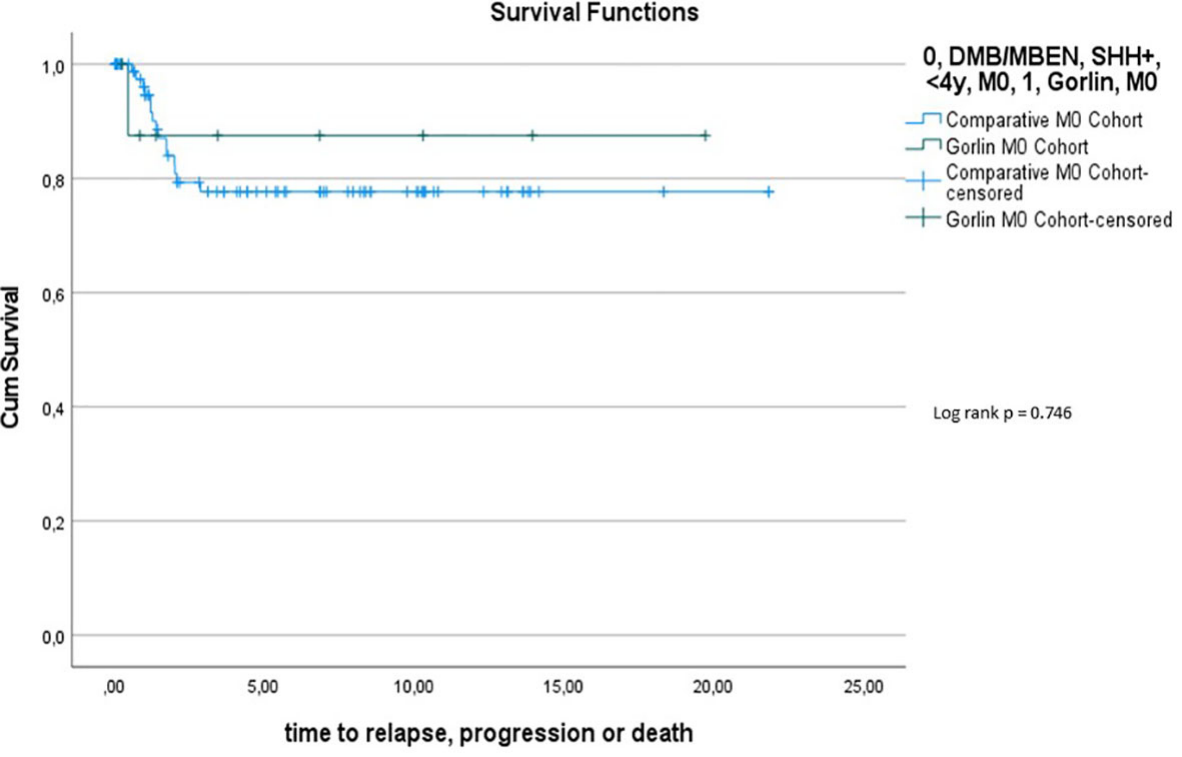
Comparison of PFS (Gorlin M0 cohort *vs*. Comparative M0 Cohort).

Comparing those same two cohorts (see **Table 2**), there was also no significant difference in terms of PFS (p=0.746): 5y-PFS in the Gorlin M0 Cohort was 87.5% ± 11.7% vs. 77.7% ± 5.1% in the Comparative M0 Cohort [see **Figure 11**) (compare to (28)].

## Discussion

Genetic testing for childhood cancer predisposition syndromes is a rapidly evolving field with significant consequences for the patient himself, the treatment regime and the prognosticated outcome as well as the affected family. Thus, whom to test and when to test will be more relevant now than ever, especially when an oncological diagnosis is first made, and a treatment plan is mapped out. To facilitate these decisions in the future we decided to retrospectively reassess the genetically diagnosed Gorlin patients in our HIT-MED database as well as look at the different patient characteristics such as age at diagnosis, histology, molecular subgroups, residual disease, staging, treatment regimens and clinical outcomes.

As shown in previous studies (55, 56), the age at diagnosis in our cohort was younger than 3.5 years with a range from 0.65 to 3.41 years. Again in line with previous publications (9, 39, 55, 56), all but one patient in our Gorlin cohort presented with medulloblastomas that were histologically classified as DMB (62.5%) or MBEN (37.5%).

In line with recommendations from other groups, nowadays we routinely suggest genetic testing for Gorlin syndrome in patients who are less than 4 years of age at the time of diagnosis and present with an SHH-activated medulloblastoma (9, 37, 57). Retrospectively, in around 1/3 (34/92 patients) of SHH-activated young MBs Gorlin syndrome was reportedly suspected and recommended to the supervising clinician. 16/34 patients (47.1%) were diagnosed with pathogenic *PTCH1* (10/16 patients) or *SUFU* variants (6/16 patients).

Since testing for SHH activation was not available for all 16 Gorlin patients (8/16 patients), we can only assume that SHH activation was present in all of them. Looking at those patients who presented with an SHH activated medulloblastoma aged 3.5 years and younger as well as assuming SHH activation for all our Gorlin patients, approximately 17% of them would have tested positive for a pathogenic *PTCH1* or *SUFU* mutation.

Since genetic testing was not conducted in all patients in our cohort, we unfortunately cannot evaluate the cumulative incidence of Gorlin syndrome. Taking into account that 16 out of 2187 patients from our cohort of patients with medulloblastomas were genetically diagnosed with Gorlin syndrome, we can only attest for these 0.7%. Judging by the low number of genetic analysis conducted within this cohort, e.g. only 4/16 cases (25%) were screened for germline variants by the MNP 2.0 study (36) in our cohort, and the fact that some medulloblastoma associated genes have only recently been described and not yet added to the standard workup for patients with SHH activated MB (19), we hypothesize that the number of undetected cases is much higher.

HIT-MED Guidance currently recommends SKK chemotherapy as initial therapy for SHH activated, low risk medulloblastomas such as DMB and MBEN in children aged 0-4/5 years (see current HIT-MED Guidance) (26, 27). SKK chemotherapy was administered in all patients initially treated in Germany (15/16) and started in the remaining patient after relocating treatment to Germany. 10/16 patients (62.5%) achieved complete remission after completing their initial treatment regimen; the 10y-OS for all Gorlin patients was 93.3% ± 6.4%. This is in line with previously described 10y-OS from our group of 82% ± 12% for Gorlin patients (55).

Radiotherapy was not administered as part of the initial therapy in any of our Gorlin patients (0/16). This is in concordance with many radiotherapy-sparing treatment regimens for young children with medulloblastomas, potentially avoiding the devastating long-term neurological effects (26–28, 35). It was however administered later on in 2 patients (12.5%); once as part of a relapse regime and once in a patient with stable residual disease after initial R+ resection. One of the two relapsed patients terminated radiotherapy after the diagnosis of Gorlin syndrome and in one other patient radiotherapy was not administered at all during disease progression because of the genetic diagnosis (see **Figure 2**).

Overall 5/16 patients (31.3%) developed additional neoplasms (see **Figure 3**): they comprised of basal cell carcinomas, odontogenic/jaw cysts, ovarian fibromas and a meningioma. These tumors mostly developed in patients who did not receive radiation at any point. While we do not know for certain when some of these tumors appeared, some were reported before the start of the treatment of the medulloblastoma. These findings question reports that postulate a higher risk for secondary malignancies in Gorlin patients after radiotherapy and suggests a generally increased risk for such entities due to the genetic diagnosis of Gorlin syndrome and/or – possibly even more likely - after the administration of chemotherapy or radiotherapy in these mutation-prone patients (41, 58–60). Because Gorlin patients present with a generally increased risk for neoplasms of up to 100% (33), we decided to title the secondary neoplasms documented in this cohort Additional Neoplasms for accuracy (see Results).

Ideally, genetic counseling should be offered to all families of young children with DMB, MBEN or SHH-activated medulloblastoma directly after diagnosis and germline testing performed in a laboratory certified for these analyses. However, not all patients are offered this opportunity or follow these recommendations. When including patients in studies like the MNP study (36), somatic variants in tumor DNA might be identified like in our patients. These patients should then be referred to undergo genetic counseling and be tested for the germline variant. Usually in Gorlin patients, these variants will be confirmed in lymphocyte DNA, like in our patients. However, there are cases where somatic *SUFU* or *PTCH1* variants were identified in tumor DNA but could not be confirmed in the germline. If these variants appear in DNA from multiple neoplasms of the same patient or the patient presents with additional features of Gorlin syndrome, mosaicism should be considered (34, 61).

Overall survival within the Gorlin patients varied depending on mutational status. Potentially at least partially owed to the shorter follow up period, *SUFU* mutated patients had a 10y-OS of 100% while *PTCH1* mutated patients had a 10y-OS of 90%. The difference between these two groups was not statistically significant (p=0.414). However, the 5y-PFS in the *SUFU* mutated cohort was 41.7% ± 22.2%, while the 5y-PFS in the *PTCH1* mutated cohort was 88.9% ± 10.5% - even though this difference again was not statistically significant (p=0.139). This is in concordance with previous studies hinting at a reduced PFS and OS for *SUFU* mutated patients compared to *PTCH1* mutated patients or SHH activated DMB/MBEN medulloblastoma patients in general (45, 57). We hypothesize that a bigger sample size and/or longer observational period might have yielded a statistically significant difference with a less favorable outcome for *SUFU* mutated patients.

To assess this further, we compared OS and PFS of Gorlin patients to SHH activated, MBEN/DMB, younger than <3.5 years at the time of diagnosis from the registries: Neither Gorlin *vs*. the Non Gorlin/Comparative Cohort (p=0.228), nor *SUFU vs*. *PTCH1* + Non Gorlin cohort (p=0.122) nor Comparative/Non-Gorlin cohort *vs*. *PTCH1 vs*. *SUFU* (p=0.302) yielded significantly different results in terms of PFS and OS.

To exclude a bias owed to metastatic disease in some of the Gorlin patients in this cohort, we created a subgroup of Gorlin patients with M0 status (Gorlin M0 cohort, n = 13). To evaluate potential differences in PFS and OS for this cohort we compared these patients to the recently published SHH-activated, M0 infant medulloblastoma cohort by Mynarek et al. who also received SKK chemotherapy (Infant MB) (28). Again, there was no significant difference in terms of OS (p=0.812) or PFS (p=0.746) between these cohorts. Also, comparing non *SUFU* and *SUFU* patients in these cohorts alone, there was not significant difference between 3y-OS (92.1% ± 3.8% *vs*. 100%; p=0.623) or 3y-PFS (86.2% ± 4.9% *vs*. 75% ± 21.7%; p=0.337). After exclusion of the potential confounding variable that is metastatic disease, these findings confirm that *SUFU* mutated patients tend to present with more events over the course of time (reduced EFS/PFS), but larger numbers and longer follow up would be needed to assess for statistically significant differences between the *SUFU* and *PTCH1* versus non Gorlin MB patients.

When assessing PFS and OS we noticed the significant disparity in follow up periods for the *PTCH1* and *SUFU* mutated cohort (12.1 years *vs*. 2.58 years). We can only assume, that a longer follow up period especially for the *SUFU* mutated cohort might have altered some of the reported differences in PFS and OS to a statistically significant level. As to why the follow up for this subgroup was so much shorter, we cannot exclude the possibility that *SUFU* associated medulloblastomas represent a new tumor entity that recently rose in numbers. But more likely we assume that this is owed to the fact that *SUFU* mutations were first described in 1999 and only a few years ago added to the list of MB/Gorlin associated genes (62). Because of this timeline, a comparative prospective analysis of *PTCH1 vs*. *SUFU* associated Gorlin patients would potentially only be valid from the year 2000 on. More contradictory still, our *SUFU* patients present with a median follow up of 2.58 years only with most *SUFU* mutations identified within the last 0-4 years, allocating the least recent *SUFU* diagnosis in our cohort to the year 2014 (4 I-HIT-MED patients, 2 patients from the Interim Registry). We hypothesize that a retrospective genetic analysis of older *PTCH1* negative, SHH activated infant MBs would potentially reveal more *SUFU* associated Gorlin patients as demonstrated by Smith et al. (2014) (47). Furthermore, recently *PTCH2* and possibly also *SMO* mutations have been described as a potential cause for Gorlin syndrome associated medulloblastoma (43, 63, 64). These genes have not (yet) been implemented in routine genetic testing strategies for patients with the suspected diagnosis of Gorlin syndrome and might (partially) close the diagnostic gap in young children with SHH-activated medulloblastomas lacking germline *PTCH1* or *SUFU* mutations.

In line with previous reports, 5 patients in our cohort presented with motor and/or global developmental delay during the follow-up period (27, 60). While temporary motor delay is often reported in children with Gorlin syndrome, persisting global developmental delay is not a common finding (31). There are few affected cases in the literature, which may be caused by more complex genetic alterations, such as the 9q22.3 microdeletion encompassing the PTCH1 gene (65). However, point mutations have not commonly been associated with global developmental delay/intellectual disability (ID) (33). While developmental delay after the treatment of medullobastoma in children with Gorlin syndrome might be a secondary effect of the treatment itself, the occurrence of developmental delay before the diagnosis of the medulloblastoma is uncommon.

Interestingly, two additional dysmorphic Gorlin patients in our cohort – one harboring a *PTCH1* and the other a *SUFU* mutation - presented with developmental delay prior to the diagnosis of the medulloblastoma/the beginning of treatment. Unfortunately, follow-up neuropsychological evaluation was not available for these 2 patients, but this finding might illustrate the need to monitor the global development of Gorlin patients closely, notwithstanding the diagnosis/treatment of a medulloblastoma.

## Conclusion

All SHH activated medulloblastoma patients younger than 4 years of age at diagnosis – especially DMB and MBEN - should be evaluated for Gorlin syndrome and systematically undergo specific genetic testing. Taking our data and previously published data on Gorlin patients into account, we can only assume that there are many Gorlin patients out there who escaped diagnosis. Since this diagnosis affects treatment, clinical management, familial planning and strongly influences the outcome, the diagnosis of Gorlin syndrome in a patient with medulloblastoma should be made as early as possible. Effective chemotherapeutic treatment strategies aiming to avoid radiotherapy during primary treatment are available, but the optimal regimen for Gorlin patients needs to be further investigated. More retrospective and prospective international studies to assess treatment, long-term survival and secondary neoplasms of the *PTCH1* and *SUFU* mutated Gorlin subgroups are warranted.

## Data Availability Statement

The data analyzed in this study is subject to the following licenses/restrictions: Privacy restrictions (patient names, family pedigree, clinical course, etc) in HIT-MED database. Requests to access these datasets should be directed to KK, k.kloth-stachnau@uke.de or katja.kloth@gmail.com.

## Ethics Statement

Ethical review and approval were not required for the study on human participants in accordance with the local legislation and institutional requirements. Written informed consent to participate in this study was provided by the participants’ legal guardian/next of kin.

## Author Contributions

KK, SR, and MM contributed to conception and design of the study. KK, MM, DO, and SR accessed the database and generated the dataset. KK performed the statistical analysis and wrote the first draft of the manuscript. SR supervised the writing of the manuscript and the design of the study. KK, MM, DO, BB, DS, TP, MW-M, and SR provided relevant data. All authors contributed to manuscript revision, read, and approved the submitted version.

## Funding

The HIT-MED trial office and the Neuroradiological Reference Center are supported by Deutsche Kinderkrebsstiftung.

## Conflict of Interest

The authors declare that the research was conducted in the absence of any commercial or financial relationships that could be construed as a potential conflict of interest.

## Publisher’s Note

All claims expressed in this article are solely those of the authors and do not necessarily represent those of their affiliated organizations, or those of the publisher, the editors and the reviewers. Any product that may be evaluated in this article, or claim that may be made by its manufacturer, is not guaranteed or endorsed by the publisher.
